# Sewerage and Agriculture
*The Application of Sewage to Agriculture. By Dugald Campbell, F.S.A. London: 1857. [Paper read before the Chemical Society.]


**Published:** 1857-07

**Authors:** 


### THE EPITOME OF SANITARY LITERATURE.

### SEWERAGE AND AGRICULTURE.*

MR. CAMPBELL gives in this essay a history of the various plans for the utilisation of sewerage, which we embody, as of value to such of our readers whose minds may now be directed to the subject.

"In 1802, a person of the name of Estienne proposed to gather the excrements into tanks, to allow the liquid parts to run off, and to dry the residue in the sun, either alone or mixed with lime.

"When this dry mass was stowed into sheds in heaps, its temperature rose to 212 deg. Fah.; afterwards it was crushed into a powder, which is stated to have been devoid of all smell, and not very bulky. A few years afterwards Sir Humphrey Davy, in a lecture delivered to the Board of Agriculture, mentioned especially mixing quicklime with night soil, to deprive it of its disagreeable smell, and observed at the same time that the Chinese, whom he considered to possess more practical knowledge of the use and application of manures than any other nation, are in the habit of mixing their night soil with one third of its weight of fat marl, make it into cakes, and dry it by exposure to the sun. 'These cakes,' Sir Humphrey adds, 'we are informed by the French missionaries, have no disagreeable smell, and form a common article of commerce of the empire.'

"After these suggestions on disinfecting night soil, and depriving it of odour, no further progress appears to have been made until as late as 1835, when M. Poitevin noticed the value of carbon, as an adjunct to marl, for disinfecting substances, and obtained a patent for the preparation of a powder containing charcoal for this purpose.

"The first after M. Poitevin appears to have been the Count de Hompesch, in 1841; he proposes to grind up the carbonaceous residue from the retorts after distilling the oils, from clay, slate, asphalte, or minerals containing bitumen, and to mix this powder with animal or putrid matters, to form a most powerful manure without any smell. The Count observes that, when iron pyrites is present in the mineral, which would be the case were schist employed, it is to be dissolved out of the powder with an acid previous to use.

"In 1842 Dominic Frick Albert proposed to collect the refuse from nearly every trade and manufacture, to mix it with animal excretion and charcoal for the purpose of making manure.

"In June, 1845, M. Du Buisson distilled oils from bituminous matters, and employed the carbonaceous residues from the retort mixed with the ammoniacal liquor from the distillate to absorb

* The Application of Sewage to Agriculture. By DUGALD CAMPBELL, F.S.A. London: 1857. [Paper read before the Chemical Society.]

fresh or coagulated blood, or any other soft animal material, such as night soil or brains, or liquid, such as urine, by which the power of the manure is increased.

"In August 1845, John Evans proposed to manufacture manure by collecting and mixing together every kind of animal refuse with urine and the refuse of a great many trades. These substances were to be treated under different circumstances with different chemical agents, such as decoctions of oak, pyroligneous acid, sulphate of iron, alum, pyrolignite of iron, naphtha mixed with a small quantity of nitric acid, sulphuric acid, and magnesia.

"Early in 1847 Mr. Edward Brown proposed to neutralise the odious and noxious gases emanating from fecal substances so as to preserve them for manure without injury to the public health by means of sulphate of iron, chlorides of sodium, iron, or manganese, nitrates, sulphates, and chlorides of lead, copper, zinc, and tin, pyroligneous acid, coal tar, or schistous and bituminous extracts; the fecal substances were to be mixed with an absorbent powder made by incinerating, in close vessels, coal or wood ashes and earth, street or road sweepings, sawdust, bonedust, and the waste water of tan yards, cotton mills, &c.

"In May 1847, Mr. William Bridges Adams and Mr. Robert Richardson proposed to construct, at railway stations, close cisterns of slate or metal, or other eligible material, into which the urine might drain, or to use chemicals for absorbing the volatile alkali and other gases.

"Edward Parker, in October 1847, proposed to submit a mixture of vegetable and mineral materials to the action of fire, and amongst the things suggested are night soil and urine.

"Mr. James Young, now of Glasgow, in a paper read before the Manchester Literary and Philosophical Society in December 1847, suggested for deodorising cesspools the refuse of the chlorine process, which is principally chloride of manganese, and which he stated was at that time a useless product, one factory throwing away daily thirty-six tons of this solution of about sp. gr. 1,280 or 1,300.

"Mr. Jasper Wheeler Rogers, in 1848, proposed to prepare charcoal from peat and combine it with animal excrement, for the purpose of making an inodorous manure.

"In 1849, M. Louis Napoleon Legras proposed to construct water-closets in such a manner as to separate and keep apart the solid fæces from the liquid. These were afterwards to be deodorised and mixed with charcoal ground to a powder, or soot.

"Mr. Henry J. Tarling, in 1850, manufactured manure with night soil and highly carbonised refuse tan as the disinfecting material.

"In June in the same year, Paul D'Angely deodorised every species of excreta by using a fluid composed of fresh bark, rue, or wild mint and sulphate of iron; the fæcal material was afterwards to be dried in a chamber and mixed with powdered peat charcoal.

"In 1850, James Hamilton Brown disinfected fæcal and putrid matters by the addition of basic salts, such as subsulphate of the protoxide of iron. A solid disinfecting agent, or one in a state of paste, was to be made by mixing 'some metallic salts with some cheap oleaginous material.' Chloride of calcium neutralised by gas liquor was to be employed likewise, or a lactiform liquid, made by mixing oil with water and some alkali. No carbonaceous matter appears to have been added; but, in case filtration were necessary, preference is given to a filter formed of carbonaceous substances. It is also stated that more rapid and perfect clarification or decantation may be effected by the addition of any compound of alumina, but particularly the double sulphate of alumina and potash, or the impure sulphate of alumina.

"In 1852, Mr. William Armand Gilbee employed a decomposing powder, composed of molasses or the residues thereof, slacked lime, sulphate of iron or zinc, and clayish magnesian earth, which was to serve the purpose of converting fecal matters into a manure fit for agricultural purposes.

"In 1854, Mr. Thornton John Herapath employed the coke of Boghead coal or Torbane mineral, either before or after the aluminous ingredients are extracted, for drying, deodorising, and absorbing urine, fæces, etc.

"In October of the same year, Mr. William White carbonised night soil or other animal refuse and fish in a close retort, and while in a state of incandescence introduced a quantity of potash or soda, then, after closing the mouth of the retort, the whole was exposed to a bright red heat for about an hour; a portion of the carbonaceous matter is said to combine with the nitrogen of the atmosphere which finds its way into the retort, forming cyanogen; this unites with the base of the alkali employed. The distillate is condensed in a solution of common salt or dilute acid, added to the cyanic compound in the retort, and the whole afterwards mixed with such substances as dried night soil, superphosphate of lime, common salt, wood ashes, dried blood, and soot. Disease, attacks of insects, or fungi on plants, are said to be prevented by a mixture of hydrate of lime, sulphate of alumina, sulphate of protoxide of iron, sulphate of magnesia, charcoal, prepared as above, biphosphate of lime, and chloride of sodium, together with the condensed volatile matters, and this is termed the 'plant preservative.'

"Mr. Alexander Macdougall, in February 1856, proposed to subject night soil and a number of other animal matters to the joint action of heat and sulphurous acid; and, in July of the same year, Mr. James Alexander Manning manufactured manures or fertilising agents from the waste of towns and other localities by subjecting fæcal matters from privies and cesspools, and all animal excrements, stale urine, also the diluted or undiluted urine from public urinals, railway stations, and factories, together with other kinds of refuse, to the action of sulphuric acid or distillation.

"Mr. Bridges Adams has, in a recent number of *The Engineer*, discussed the possibility of dealing with the excrement of the population of London without allowing it to pass into the sewers, He considers that if, by a mechanical arrangement of water-closets, the fæces and urine are collected apart, the latter may be barrelled up and very profitably transported by rail to agricultural districts."

The value of manurial substances, contained in human excreta, is estimated by our author thus :—

"For a population of 10,000, the quantity of dry solid in the excrement yearly will amount to 205 tons, which would have a money value of £3,075 sterling. This estimate is made upon the assumption that the ammonia in excremental material is worth £70 per ton; the phosphoric acid £56 per ton; and the potash £70 per ton.

"The chief element of the manurial value of town sewage is the excremental material, and this, in the instance of London, with a population of 2,600,000, amounts annually to 53,393 tons of dry solid, which, as I have already shown, contains ingredients which give it a value of £15 per ton at least.

| | |
|---|---|
| The quantity of ammonia which this contains or is capable of producing is .. .. .. .. .. .. | 11,440 tons. |
| The phosphoric acid .. .. .. .. .. .. | 1,839 „ |
| And the potash, etc. .. .. .. .. .. .. | 1,331 „ |

These three items make up a money value of about £836,834.

"The number of methods that have been proposed for obtaining manure from town sewage are not by any means so numerous as those for working excremental substances. For the present purpose they may be considered under three heads :—

"1st. Filtration through various media, before, and after the addition of chemical substances.

"2nd. Precipitation by means of various reagents.

"3rd. Irrigation."

Reviewing the three modes here named, Mr. Campbell inclines to that by irrigation; and concludes, that by no process of chemistry hitherto known, can a highly valuable solid manure be obtained from town sewerage alone.

